# Examining the needs, outcomes and current treatment pathways of 2461 people with treatment-resistant depression: mixed-methods study

**DOI:** 10.1192/bjp.2024.275

**Published:** 2026-02

**Authors:** Kiranpreet Gill, Danielle Hett, Max Carlish, Rebekah Amos, Ali Khatibi, Isabel Morales-Muñoz, Steven Marwaha

**Affiliations:** 1 Institute for Mental Health, School of Psychology, University of Birmingham, Birmingham, UK; 2 The Barberry, National Centre for Mental Health, Birmingham and Solihull Mental Health NHS Trust, Birmingham, UK

**Keywords:** Electronic health records, mixed methods, major depressive disorder, treatment-resistant depression, treatment pathway

## Abstract

**Background:**

A substantial subset of patients with major depressive disorder (MDD) experience treatment-resistant depression (TRD), typically defined as failure to respond to at least two sequential antidepressant trials at adequate dose and length.

**Aims:**

To examine clinical and service-level associations of TRD, and the experiences of people with TRD and clinicians involved in their care within a large, diverse National Health Service trust in the UK.

**Method:**

This mixed-methods study integrated quantitative analysis of electronic health records with thematic analysis of semi-structured interviews. Chi-squared tests and one-way analysis of variance were used to assess associations between lines of antidepressant treatments and sociodemographic and clinical variables, and binary logistic regression was used to identify associations of TRD status.

**Results:**

Nearly half (48%) of MDD patients met TRD criteria, with 36.9% having trialled ≥4 antidepressant treatments. People with TRD had higher rates of recurrent depression (odds ratio = 1.24, 95% CI: 1.05–1.45, *P* = 0.008), comorbid anxiety disorders (odds ratio = 1.21, 95% CI: 1.03–1.41, *P* = 0.019), personality disorders (odds ratio=1.35, 95% CI: 1.10–1.65, *P* = 0.003), self-harm (odds ratio = 1.76, 95% CI: 1.06–2.93, *P* = 0.029) and cardiovascular diseases (odds ratio = 1.46, 95% CI: 1.02–2.07, *P* = 0.0374). Greater treatment resistance was linked to increased economic inactivity and functional loss. Qualitative findings revealed severe emotional distress and frustration with existing treatments, as well as organisational and illness-related barriers to effective care.

**Conclusions:**

TRD is characterised by increasing mental and physical morbidity and functional decline, with individuals experiencing barriers to effective care. Improved pathways, service structures and more effective biological and psychological interventions are needed.

## Defining and understanding treatment-resistant depression

Major depressive disorder (MDD) is a pervasive, debilitating condition that has a significant impact on quality of life,^1^ leading to disability,^2^ physical and mental health comorbidities,^3^ and increased mortality.^4,5^ Treatment-resistant depression (TRD), a subset of MDD in which existing treatments fail to alleviate symptoms, affects approximately one-third of individuals.^6,7^ Definitions of TRD vary, impeding generalisability and comparison of research findings between studies.^8,9^ To address this, we have adopted a pragmatic approach and define TRD as failure to respond to at least two sequential trials of antidepressants at adequate dose and length, aligning with common practices.^8,9^

The mental, physical and social impact of TRD exceeds that of MDD; thus, it represents one of the most challenging yet neglected conditions within psychiatry.^10,11^ Most research has relied on large cohort studies using electronic health records (EHRs), with few studies having used mixed-methods approaches that include patient and clinician perspectives. So far, three UK cohort studies have leveraged anonymised EHR data to examine treatment journeys and outcomes of people with TRD.^11–13^ Consistent with previous research,^14–17^ all three of these studies highlighted the disproportionately severe consequences of TRD, with two reporting suboptimal treatment outcomes for people with TRD,^12,13^ deviating from the recommended stepwise approach.^18^

Qualitative studies have revealed mutual challenges faced by people with TRD and clinicians, including dissatisfaction with treatment options and ambiguous treatment guidelines.^19–22^ This results in individuals feeling that their depression has become integral to their identity, owing to unclear explanations and limited treatment alternatives.^21,22^ As the considerable impact of TRD becomes increasingly apparent, this study addresses a major research gap by gathering perspectives from both people with TRD and clinicians to better understand their needs. These perspectives will be vital in helping shape effective interventions.

## Aims and mixed-methods approach

This is the first study to adopt a mixed-methods approach to address these gaps. We aim to provide valuable insights into TRD by (a) identifying and estimating its prevalence; (b) comparing sociodemographic, clinical and service use outcomes between people with TRD and those with MDD; (c) examining outcomes by level of treatment resistance; and (d) exploring treatment experiences within a large UK secondary care National Health Service (NHS) trust offering a range of mental healthcare services across a diverse population of 1.3 million people in Birmingham and Solihull.^23^ Previous research has predominantly relied on quantitative data, which fails to capture the multifaceted nature of TRD.^11–13^ Our mixed-methods approach combines quantitative analysis with qualitative exploration of individual narratives, capturing population characteristics, outcomes and lived experiences. Understanding these dimensions is crucial for identifying treatment gaps and developing tailored interventions, compassionate care strategies, and policies to address the substantial global burden of TRD.

## Method

### Quantitative study

#### Data source

EHRs routinely maintained by the NHS were used to identify current patients diagnosed with depression up to 2021, with some diagnoses dating back to 1996. We focused on people actively currently receiving care to understand ongoing treatment challenges in this population. The information team of the NHS trust anonymised and extracted these records through a retrospective audit of de-identified EHRs, based on the criteria outlined below. Data were analysed using Microsoft Excel and SPSS version 29.

#### Study cohort

Eligible individuals identified in the audit were aged ≥18 years with a diagnosis of MDD ICD-10 codes F32–F33, indicating current or recurrent episodes. Those with comorbid diagnoses (current or historical) of (a) bipolar disorder and/or mania or of (b) psychosis-related, (c) dementia-related or (d) cognitive and/or neurological disorders (similar to past research^15^), as well as those without a history of antidepressant use, were excluded.

The Maudsley Prescribing Guidelines, a widely recognised resource for managing complex cases of depression, including TRD, were used to classify people with TRD.^24^ These guidelines align with the recommendations of the British Association for Psychopharmacology on treatment strategies for resistant depression.^25^ TRD was defined as failure to respond to at least two antidepressants prescribed at a therapeutic dose for 4–6 weeks within the current depressive episode, with treatment progressing to a third antidepressant and/or an augmenting agent (e.g. lithium) following two failed trials. Those meeting these criteria were classified as having TRD, whereas others were classified as having MDD.

#### Data extraction

After identifying MDD patients using ICD-10 codes from structured EHR fields, data extraction involved two stages. In stage 1, people with TRD versus MDD were classified using Electronic Prescribing and Medicines Administration data within secondary care. People with TRD were identified by progression to a third antidepressant or the addition of an augmenting agent following inadequate response to two prior antidepressant trials. In stage 2, service use and clinical referral data were extracted across all mental health services within the NHS trust, including community, urgent care, specialist, in-patient, forensic and addiction services (Supplementary Fig. 1, available online at https://doi.org/10.1192/bjp.2024.275). These services cover the full spectrum of depression care, allowing for detailed mapping of treatment journeys.

Antidepressant prescriptions (including augmentation drugs), along with sociodemographic data (age, gender, ethnicity, employment status) and clinical characteristics (ICD-10 diagnosis codes, mental and physical health comorbidities) were also collected for analysis. See the Supplementary Material for further details.

#### Statistical analysis

Descriptive statistics, frequencies and percentages were calculated for antidepressant prescriptions, sociodemographic data, clinical comorbidities since depression diagnosis and service use across six clinical services, Data were divided into two subgroups – MDD and TRD – and differences were examined using chi-squared tests for categorical variables and independent *t*-tests for continuous variables.

Additional chi-squared tests were used to assess associations of the number of antidepressant treatment lines with sociodemographic and clinical characteristics. One-way analysis of variance with Bonferroni *post hoc* comparisons was used to determine differences in age at diagnosis and employment status across different treatment lines.

Binary logistic regressions were used to investigate independent sociodemographic and clinical variables associated with TRD status using a stepwise approach across four models (Supplementary Table 1). Each model included specific predictor variables chosen based on their significance and observed group differences (as outlined in Tables 1 and 2). Model 1 included sociodemographic and clinical variables (depression ICD-10 code at first diagnosis, psychotic illness, and employment status). Model 2 added mental health comorbidities (substance misuse, anxiety and personality disorders). Model 3 integrated recorded history of deliberate self-harm. Model 4 included physical health comorbidities (cardiovascular, respiratory, gastrointestinal diseases).

**Table 1:** Sociodemographic characteristics by depression status

Sociodemographic characteristics	MDD		TRD		*P*-value
	*N* (%)	Mean (s.d.)	*N* (%)	Mean (s.d.)	
F32 ICD-10 diagnosis code (all)^a^	1963 (73.38)	–	1679 (68.22)	–	**<0.001**
F33 ICD-10 diagnosis code (all)^a^	712 (26.62)	–	782 (31.76)	–	**<0.001**
F32/F33 ICD-10 diagnosis with psychotic symptoms reported (i.e. ICD-10 codes F32.2 and F33.3)^b^	222 (8.30)	–	370 (15.03)	–	**<0.001**
Age in years at earliest recorded depression diagnosis in the trust^c^	16.53 (44.09)	–	15.48 (43.87)	–	0.349
Age at point of data extraction^d^	–	49.28 (16.56)	–	49.24 (15.53)	0.470
Reported deaths at point of data extraction^e^	193 (7.20)	–	140 (5.70)	–	**0.026**
Age in years at reported death	–	65.41 (17.62)	–	60.10 (16.00)	–
Ethnicity^f^	–	–	–	–	**0.057**
Black	139 (5.20)	–	99 (4.02)	–	–
Mixed	76 (2.54)	–	69 (2.60)	–	–
South Asian	441 (16.49)	–	416 (16.90)	–	–
White	1818 (67.96)	–	1729 (70.26)	–	–
Unknown/not reported	201 (7.51)	–	148 (6.01)	–	–
Employment status	–	–	–	–	**<0.001**
Employed	335 (12.52)	–	322 (13.08)	–	0.545
Unemployed (seeking)	234 (8.74)	–	233 (9.47)	–	0.374
Economically inactive	872 (32.60)	–	1015 (41.24)	–	0.372
Unknown/not reported	1234 (46.13)	–	891 (36.20)	–	**<0.001**

MDD, major depressive disorder; TRD, treatment-resistant depression.

a. *χ*^2^ = 16.54(1), *P* < 0.001.

b. *χ*^2^ = 57.02(1), *P* < 0.001.

c. *t*(5134) = 0.349, *P* = 31.

d. *t*(5134) = 0.07, *P* = 0.470. Point of data extraction was October 2021.

e. *χ*^2^ = 4.92(1), *P* = 0.026. Point of data extraction was October 2021.

f. *χ*^2^ = 9.17(4), *P* = 0.057.

**Table 2:** Mental and physical health comorbid diagnoses by depression status

	MDD (*N* = 2675)	TRD (*N* = 2461)		
Diagnoses	*N* (%)	*N* (%)	*P*-value	*χ*^2^ (1) (MDD versus TRD)
Comorbid mental health diagnoses				
Substance misuse disorders, including misuse and dependence (yes; at least one of the below disorders)	486 (18.17)	513 (20.85)	**0.015**	5.86
Alcohol	252 (9.42)	226 (9.18)	0.770	0.086
Drugs of misuse	60 (2.24)	73 (2.97)	0.103	2.66
Sedatives or hypnotics	4 (0.15)	10 (0.41)	0.078	3.11
Tobacco	123 (4.60)	170 (6.91)	**<0.001**	12.71
Other and/or multiple drugs	47 (1.75)	34 (1.38)	0.271	1.21
Anxiety disorders (yes; e.g. generalised anxiety, post-traumatic stress, social anxiety, panic disorder)	656 (24.50)	759 (30.80)	**<0.001**	25.63
Eating disorders (yes; e.g. anorexia, bulimia)	50 (1.87)	39 (1.58)	0.435	0.61
Personality disorders (yes; e.g. borderline personality disorder)	299 (11.18)	407 (16.54)	**<0.001**	31.06
Self-harm	25 (0.90)	66 (2.70)	**<0.001**	22.48
Psychotic illness	222 (8.30)	370 (15.03)	**<0.001**	57.02
Developmental disorders (yes; e.g. Asperger’s, autism)	42 (1.57)	44 (1.79)	0.543	0.369
Comorbid physical health diagnoses				
Diabetes	48 (1.79)	63 (2.56)	0.059	3.55
Type 1 diabetes	10 (0.376)	16 (0.653)	0.163	1.94
Type 2 diabetes	31 (1.16)	45 (1.83)	**0.047**	3.94
Unspecified diabetes	7 (0.26)	45 (1.83)	0.443	0.60
Cardiovascular diseases (yes; e.g. hypertension, ischaemia, diseases of veins)	89 (3.33)	135 (5.49)	**<0.001**	14.32
Respiratory diseases (yes; e.g. asthma, bronchitis, pneumonia)	74 (2.77)	93 (3.78)	**0.041**	4.18
Gastrointestinal diseases (yes; e.g. irritable bowel syndrome, ulcers, liver disease)	63 (2.36)	118 (4.79)	**<0.001**	22.44

MDD, major depressive disorder; TRD, treatment-resistant depression.

### Qualitative study

#### Research team

The qualitative study was developed with the patient and public involvement and engagement (PPIE) group of the NHS trust, Lived Experience Action Research (LEAR); the group was chaired by M.C., who has extensive lived experience of TRD.

#### Eligibility criteria

Eligible individuals were those receiving treatment at the NHS trust who met the widely accepted TRD criteria, defined as failure to respond to at least two trials of antidepressant medication at adequate dose and length.^9^ We excluded individuals with comorbid mental, cognitive or neurological conditions.^11^

#### Clinicians

Eligible clinicians were directly involved in the care of people with TRD.

#### Recruitment

Participants were purposely recruited from the NHS trust through internal channels, with support from M.C. and members of LEAR. We sought variation in participant characteristics including duration of TRD and clinician background or specialty. Interested clinicians contacted the research team. Eligible people with TRD responded to participant-facing flyers or were informed by their care coordinators, who then shared their details with the research team.

#### Data collection

Two interview schedules were produced in collaboration with the PPIE group, covering experiences of living with or treating TRD, views on current treatments and suggestions for future treatment options (see the Supplementary Material for full schedules). Following provision of informed consent, interviews were conducted face-to-face or virtually based on participant preference and recorded with encrypted devices with permission. Interviews lasted for 30–60 min and were conducted by three researchers. Each researcher transcribed their interviews.

#### Analysis

Qualitative data were analysed using thematic analysis,^26^ adopting a pragmatic epistemology to complement quantitative data. Six steps were undertaken: (a) familiarisation with transcripts, (b) line-by-line coding, (c) construction of themes, (d) review of themes, (e) definition and naming of themes, and (f) reporting with relevant quotes, with clinicians referred to as ‘C1’ etc. and people with TRD referred to as ‘P1’ and so on. The analysis focussed on understanding perspectives, experiences and treatment needs of people with TRD, along with clinicians’ views. Researchers maintained reflexivity, acknowledging their mental health backgrounds and potential biases; these were addressed through post-interview debriefs. Regular team discussions ensured reliability and consensus on interpretations among researchers. Intensive coding facilitated data saturation (*N* = 15), indicating that additional interviews would provide minimal new information.^27^

#### Ethical considerations

All participants provided written informed consent before interviews. The authors assert that all procedures contributing to this work comply with the ethical standards of the relevant national and institutional committees on human experimentation and with the Helsinki Declaration of 1975, as revised in 2013. This study was approved as a service evaluation by the Research and Development Department of the Birmingham and Solihull Mental Health NHS Foundation Trust (ref. SE0287), following the trust’s guidelines.

## Results

### Quantitative findings

#### Prevalence of TRD at the NHS Trust

Of the 5136 patients diagnosed with MDD without exclusionary mental health comorbidities, 2461 (47.92%) met TRD criteria, and the remaining 2675 (52.08%) were classified as having MDD.

#### Sociodemographic characteristics and clinical outcomes by TRD status

Table 1 summarises sociodemographic and clinical variables by TRD status. Although single episodes of MDD occurred in both groups, people with TRD exhibited significantly higher prevalence of recurrent depression compared with those with MDD (31.76% *v*. 26.62%, *χ*^2^(1) = 16.54, *P* < 0.001). Economic inactivity was more common in people with TRD (41.24% *v*. 32.60%, *P* < 0.001, adjusted for multiple testing). No significant differences were found for age at diagnosis (*F*(3, 5132) = 2.41, *P* = 0.065). However, although the proportion of deaths was lower among people with TRD (5.70% *v*. 7.20%) compared with MDD patients (*χ*^2^(1) = 4.92, *P* = 0.026), individuals with TRD who died were, on average, approximately 5 years younger at time of death than those with MDD (*t*(331) = 2.82, *P* = 0.003). Table 2 presents clinical data on both mental and physical health comorbidities by TRD status. Compared with those with MDD, people with TRD had significantly higher rates of substance use (20.85% *v*. 18.17%, *P* = 0.015), anxiety disorders (30.80% *v*. 24.50%, *P* < 0.001), personality disorders (16.54% *v*. 11.18%, *P* < 0.001), self-harm (2.70% *v*. 0.90%, *P* < 0.001) and psychotic illness (15.03% *v*. 8.30%, *χ*^2^(1) = 57.02, *P* < 0.001). People with TRD also showed higher prevalence of smoking-related diagnoses (6.91% *v*. 4.60% for MDD, *P* < 0.001), as well as cardiovascular (5.49% *v*. 3.33%, *P* < 0.001), respiratory (3.78% *v*. 2.77%, *P* = 0.04) and gastrointestinal diseases (4.79% *v*. 2.36%, *P* < 0.001). Similarly, people with TRD had significantly higher rates of diabetes (8.41% *v*. 5.46%, *P* < 0.001), with specific increases observed for both type 1 (1.67% *v*. 1.12%, *P* = 0.070) and type 2 (5.89% *v*. 3.85%, *P* < 0.001) diabetes.

#### Service use by TRD status

Examination of service use data showed several significant associations in referral patterns between MDD patients and people with TRD (*N* = 9161) across services (Supplementary Fig. 2). People with TRD had fewer referrals compared with MDD patients to community mental healthcare services (40% TRD *v*. 45% MDD, *P* < 0.001), namely community mental health teams (CMHTs), liaison psychiatry and home treatment teams. Instead, they were more frequently referred to specialist services (20% *v*. 15%, *P* < 0.001) such as psychology, psychotherapy and electroconvulsive therapy. People with TRD also had more referrals for in-patient services (5% *v*. 3%, *P* < 0.001), both short- and long-term, but fewer referrals to forensic services compared with MDD patients (2% *v*. 3.5%, *P* < 0.001).

#### Sociodemographic and clinical predictors of TRD status

Binary logistic regressions revealed the following independent significant associations of TRD status in the full and final model (Table 3): presence of psychotic illness (odds ratio = 1.59, 95% CI: 1.27–2.00, *P* < 0.001), single depressive episode versus recurrent depression ICD code (odds ratio = 1.24, 95% CI: 1.05–1.45, *P* = 0.008), comorbid anxiety disorders (odds ratio = 1.21, 95% CI: 1.03–1.41, *P* = 0.019), comorbid personality disorders (odds ratio = 1.35, 95% CI: 1.10–1.65, *P* = 0.003), reported self-harm (odds ratio = 1.76, 95% CI: 1.06–2.93, *P* = 0.029) and comorbid cardiovascular diseases (odds ratio = 1.46, 95% CI: 1.02–2.07, *P* = 0.037).

**Table 3:** Binary regression analysis of predictors of TRD^a^

Independent variable	**Model 1**^**b**^			**Model 2**^**c**^			**Model 3**^**d**^			**Model 4**^**e**^		
	OR	95% CI	*P*-value	OR	95% CI	*P*-value	OR	95% CI	*P*-value	OR	95% CI	*P*-value
Psychotic symptoms (no versus yes)	1.56	1.25–1.95	**<0.001**	1.64	1.31–2.05	**<0.001**	1.64	1.31–2.05	**<0.001**	1.59	1.27–2.00	**<0.001**
ICD-10 depression code (F32 versus F33)	1.24	1.06–1.45	**0.007**	1.22	1.04–1.43	**0.013**	1.22	1.04–1.43	**0.012**	1.24	1.05–1.45	**0.008**
Anxiety disorders (no versus yes)	–	–	–	1.24	1.06–1.45	**0.007**	1.23	1.05–1.44	**0.010**	1.21	1.03–1.41	**0.019**
Personality disorders (no versus yes)	–	–	–	1.39	1.15–1.70	**<0.001**	1.34	1.10–1.64	**0.004**	1.35	1.10–1.65	**0.003**
Reported self-harm (no versus yes)	–	–	–	–	–	–	1.89	1.14–3.13	**0.002**	1.76	1.06–2.93	**0.029**
Comorbid cardiovascular diseases	–	–	–	–	–	–	–	–	–	1.46	1.02–2.07	**0.037**
Nagelkerke *R*^2^	1.1%			2.2%			2.5%			3%		

OR, odds ratio; TRD, treatment-resistant depression; MDD, major depressive disorder.

a. All predictor variables were significantly different between groups (TRD versus MDD); see Supplementary Table 1 and Tables 1 and 2.

b. Model 1 included type of depression, presence of psychotic symptoms and employment status.

c. Model 2 incorporated mental health comorbidities.

d. Model 3 introduced history of self-harm.

e. Model 4 included physical health comorbidities.

#### Treatment resistance in the TRD cohort

Numbers of antidepressant prescriptions, indicating varying levels of treatment resistance, were categorised as two, three or at least four antidepressants prescribed within the current episode. In the TRD cohort, 946 people (35.40%) had trialled two lines of antidepressants, 529 (19.78%) had trialled three and 986 (36.93%) had trialled four or more (Supplementary Table 2). The total number of antidepressant prescriptions in this group was 9478.

#### Sociodemographic characteristics and clinical outcomes by treatment resistance

A significant association was observed between higher antidepressant use (three or at least four lines) and recurrent depression (F33) in people with TRD (*χ*²(3) = 22.56, *P* < 0.001). Older individuals were more likely to have tried four or more lines of treatment compared with three lines (*P* = 0.009), with a significant difference in age across levels of treatment resistance (*F*(3,5132) = 3.52, *P* = 0.014). Economic inactivity increased with treatment resistance (*F*(3,5132) = 8.65, *P* < 0.001), especially among those who had tried four or more lines compared with two (*P* < 0.001) or three lines (*P* = 0.034), and was significantly associated with number of treatment lines (*χ*²(3)=11.77, *P* = 0.008).

Although no significant associations were found between number of antidepressant treatment lines and mortality, greater treatment resistance was associated with several health conditions, including smoking-related, cardiovascular, respiratory and gastrointestinal problems. Notably, tobacco use showed a particularly strong link to higher levels of treatment resistance (*χ*²(3) = 89.56, *P* < 0.001). Psychiatric comorbidities, including substance use (*χ*²(3) = 42.11, *P* < 0.001), anxiety disorders (*χ*²(3) = 91.00, *P* < 0.001), personality disorders (*χ*²(3) = 75.81, *P* < 0.001), self-harm (*χ*²(3) = 53.21, *P* < 0.001) and psychotic illness, increased with number of antidepressant trials, particularly in those who had tried three or at least four lines of treatment (Supplementary Table 3).

### Qualitative findings

In total, 15 semi-structured interviews were conducted, involving eight clinicians (two psychiatrists, two clinical psychologists, one psychotherapist, one physiotherapist and two nursing staff) and seven people with TRD (two males, five females), aged 28 to 63 years. Six main themes emerged: (a) TRD classification criteria, (b) experiences of living with or treating TRD, (c) current treatment pathway, (d) treatment barriers, (e) treatment facilitators and (f) future treatment recommendations. Each theme included two sub-themes – see Supplementary Table 4 for details and quotes from participants.

#### Theme 1: TRD criteria

A limited understanding of TRD as an indicator of treatment failure emerged as a focal point. People with TRD frequently lacked awareness of TRD as a marker of severity and resistance to treatment, whereas clinicians used inconsistent terminology, such as chronic or recurrent depression, complicating classification and treatment. This unfamiliarity extended to the practical application of treatment guidelines, leading to frustration and hindering effective treatment planning. C5 stated, ‘I’m not even sure of pathways for depression within secondary care that are not diagnosis-specific … they should be made more explicit’.

#### Theme 2: experiences

This theme reflected the emotional impact on people with TRD and clinicians, revealing the complex challenges of managing TRD. People with TRD described how the condition affected their lives, relationships and daily functioning, including years lost to inactivity. Clinicians expressed feelings of helplessness when faced with severely distressed people with TRD and family members. Acknowledging the variable continuum of severity of TRD, both groups emphasised the need for a holistic, patient-centred treatment approach, as the ‘one size fits all’ approach, typically characterised by an overreliance on pharmacological solutions, was recognised as inadequate. P1 stated, ‘I’ve taken that many antidepressants that my synapses are just frazzled’.

#### Theme 3: current treatment pathway

Both groups discussed experiences with current treatment pathways (biological and psychological) for TRD. Some reported positive outcomes with specific antidepressant treatments, but others mentioned side-effects (e.g. poor sleep, headaches), that ‘outweighed any benefits’ (P1). There was a call for exploration of psychological interventions such as compassion-focused therapy alongside cognitive–behavioural therapy (CBT) as alternatives or adjuncts to pharmacological approaches. Both groups emphasised the importance of tailoring treatment plans to individual needs, with C7 suggesting, ‘I think we need to have different conversations around what care would patients like and what care would help them’.

#### Theme 4: barriers to treatment

Barriers to treating TRD included illness-related attitudes and institutional and/or organisational challenges, such as limited access to psychological interventions and inconsistent treatment approaches. People with TRD described treatment experiences as a ‘trial and error’ process, discouraging them from seeking further support. P6 stated, ‘my doctor doesn’t know what to do with me’. Clinicians noted that depression lacked dedicated funding and care pathways, unlike other conditions such as psychosis. C4 stated, ‘depression is singled out’. These challenges hinder both individuals with TRD and clinicians in navigating treatment options and providing clear and effective solutions, respectively; this indicates a need for improved access to TRD-specific resources and support services.

#### Theme 5: facilitators of treatment

People with TRD emphasised the importance of feeling heard by their healthcare team and appreciated initiatives to improve TRD awareness. However, discontinuity in care remained a significant issue, as expressed by P4: ‘I’m left in limbo … I’ve been here nearly four years’. Both groups advocated noting TRD prominently in patient records to streamline discussions during appointments and improve outcomes.

#### Theme 6: future treatment pathway recommendations

The final theme centred on future treatment recommendations for establishing a dedicated TRD care pathway within the NHS trust. Key recommendations included the following:

- providing tailored information for people with TRD (e.g. pamphlets) on prevalence and current treatment options;
- establishing standardised pathways to signpost people with TRD to specialised services;
- improving access to diverse psychological treatments, including CBT;
- enhancing clinicians’ awareness of and training on TRD using current research and information on TRD-specific guidelines;^24,25^
- introducing low-intensity, high-frequency forms of support such as peer groups, support workers and occupational therapy, contrary to current treatment provision;
- adopting a holistic treatment approach with consistent clinician input;
- providing increased clinical research opportunities for people with TRD.

## Discussion

Major depression presents a significant global public health challenge, particularly for those unresponsive to first-line treatments.^28^ Our mixed-methods study combined quantitative analysis and qualitative interviews to explore the clinical characteristics, experiences and treatment pathways of people with TRD, alongside clinician perspectives. Among the 5136 MDD patients, nearly 48% met TRD criteria, with 36.93% having trialled four or more antidepressant treatments. Consistent with previous research,^29–37^ people with TRD in this group exhibited more severe clinical profiles, highlighting the multifaceted nature of the condition. The qualitative findings reinforced this, illustrating the cumulative burden of repeated treatment failures.

The prevalence of TRD within secondary care in this geographical location exceeded estimates reported across other areas in the UK,^11–13^ underscoring the substantial burden of TRD in secondary care and the challenges of standardising its classification across the UK. Establishing a uniform definition for TRD in clinical practice and research is essential for enhancing comparability and understanding of the condition.

Qualitative interviews with people with TRD and clinicians highlighted difficulties in defining and understanding TRD in clinical practice. Although our study focused on TRD, we acknowledge the concept of difficult-to-treat depression (DTD) – that is, depression that remains burdensome despite standard treatment – and advocate a broader, holistic management approach.^8^ Recent DTD guidelines recommend individualised care that extends beyond symptom relief, focusing on overall psychosocial functioning and quality of life.^8^ Although TRD and DTD differ in terms of their criteria, both are complex depressive disorders that require personalised, holistic treatment.

Mental and physical health comorbidities, including cardiovascular diseases and economic inactivity, were associated with TRD. Depression nearly doubles the likelihood of concurrent mental health conditions such as anxiety, intensifying treatment resistance^37–40^ and reducing treatment efficacy.^41–43^ People with TRD who had trialled four or more antidepressants demonstrated greater clinical complexity, with higher rates of comorbid substance use and anxiety and personality disorders. This suggests that escalating resistance is associated with increasingly complex mental health challenges, with each failed treatment compounding severity. Multimorbidity worsens TRD owing to limited and sometimes ineffective treatment options, leading to reliance on antidepressants, as revealed in qualitative interviews. Patients often perceive antidepressants as their sole option;^44^ this reflects the treatment-resistant nature of TRD, which affects work, relationships and independence and further exacerbates the socioeconomic effects of the condition. Moreover, the strong link between higher treatment resistance and economic inactivity indicates functional decline in patients trialling multiple medications, reinforcing the need for interventions that address both clinical and socioeconomic aspects of TRD.

Substance use was significantly associated with TRD, complicating the relationship between poor mental health, substance use and adverse outcomes. The literature shows a strong link between depression and substance use, with people with TRD at heightened risk of substance use disorders compared with those with MDD.^45^ Substance use often serves as a coping mechanism for negative affect.^46,47^ We found that increased treatment resistance was linked to higher rates of self-harm; this reflects the substantial psychological burden of TRD and is perhaps linked with ineffectiveness of treatments. Qualitative data supported this, with many participants expressing hopelessness after repeated treatment failures and worsening mental health outcomes.

In qualitative interviews, people with TRD emphasised the need for post-discharge support; however, quantitative findings indicated lower rates of referral to community-based services such as CMHTs compared with MDD patients. Instead, people with TRD were more frequently referred to specialised and in-patient services, indicating a gap in continuous, non-urgent care outside acute settings. This lack of community-based care may complicate TRD management further. Although the reasons for the lower referral rates remain unclear, it is possible that TRD requires more specialised care beyond that which CMHTs may feel they can offer. Longer depression episodes have also been associated with treatment delays, extended time before switching medications and increased resistance.^45,48–50^ The low rate of referrals to community-based services, coupled with ineffective early-phase treatment options, may worsen the burden of TRD. This indicates a need for effective interventions during early stages of care.

### Strengths and limitations

Our mixed-methods approach combined quantitative analysis of EHRs with qualitative interviews offering comprehensive insights into TRD. The quantitative data offered robust findings with respect to TRD prevalence and clinical associations, enabling analysis based on number of treatment lines to obtain a more nuanced understanding of the severity of resistance and its impact on outcomes. The qualitative interviews captured valuable perspectives from both people with TRD and clinicians, making this the first study to explore the lived experiences of such individuals within secondary care in the UK. As our findings were based on a large, diverse data-set (*N* = 5136 for MDD, *N* = 2675 for TRD), they are applicable across varied demographics, which is crucial for tailored interventions. Collaboration with the NHS trust’s PPIE group further aligned the study with the lived experiences and treatment preferences of those affected by TRD, enhancing its practical relevance.

Despite its strengths, our study has some limitations. First, the data-set, which was primarily from secondary care prescribing records, lacked integration with primary care data; this limited our insight into treatment resistance progression before referral and potentially led to underestimation of physical comorbidities. As EHRs in mental health services focus on psychiatric diagnoses and broader ICD-10 categories, physical health conditions may be underreported. In addition, the low rates of self-harm observed in our study are likely to reflect limitations in data extraction rather than true prevalence. As we extracted data from structured fields coded using ICD-10 diagnostic codes, incidents of self-harm, which are often recorded in free-text clinical notes, were harder to capture. Therefore, the reported self-harm figures are probably underestimates of self-harm in this sample. Future research integrating primary care data and applying NLP algorithms would provide a more comprehensive understanding of TRD and its associations.

In addition, although our cohort reflected typical secondary care patients, focusing on non-prescribing clinicians in our qualitative analysis may have influenced perspectives, as prescribers, particularly psychiatrists, may have a deeper familiarity with the term TRD. This could have affected the generalisability of our qualitative findings across different clinical roles. Last, the retrospective nature of the quantitative analysis (1996 to 2021) introduced potential variability in reporting practices over time; thus, we urge caution in interpreting longitudinal trends and associations.

### Implications

In this study, we highlight the complexity of managing TRD in secondary care and advocate personalised, innovative and holistic treatment strategies beyond a ‘one-size-fits-all’ approach. Individuals with greater treatment resistance face more severe depression, with more comorbidities and functional decline, indicating inadequacy of current antidepressant trials.

Clinicians should be aware of current TRD classification, as understanding of nuanced presentations is crucial for informed decision-making and for enhancing patient engagement and treatment adherence. The TRD marker – failure to respond to at least two sequential antidepressants – should alert clinicians to the increased morbidity of this group, prompting consideration of new strategies. The link between treatment resistance and economic inactivity further indicates a need to integrate vocational and long-term functional support into TRD management. Health and social care systems must address not only medical but also psychosocial and economic challenges faced by people with TRD, ensuring that care pathways are adaptive to evolving patient needs.

In sum, this mixed-methods study highlights the urgent need for a shift in TRD management within secondary care. The high prevalence and complexity of TRD requires comprehensive, individualised approaches that address both clinical and psychosocial challenges. Researchers, clinicians and people with TRD need a more defined care pathway for this group. Access to specialised services and innovative biological and psychological therapies are essential for effectively addressing the care challenge.

## Supporting information

Gill et al. supplementary material Gill et al. supplementary material

## Data Availability

The data were de-identified and used in a data secure format, and all patients within the NHS trust have the choice to opt out of their anonymised data being used. Approval for data access can only be provided by the Research and Innovation Department at Birmingham and Solihull Mental Health Foundation Trust.
