# Supplementary material for: Examining the needs, outcomes and current treatment pathways of 2461 people with treatment-resistant depression: mixed-methods study
Source: Br J Psychiatry. 2025 Mar 12;228(2):108–15. doi: 10.1192/bjp.2024.275 (PMC12823348; doi:10.1192/bjp.2024.275)
Supplement: Gill et al. supplementary material [file S0007125024002757sup001.docx]

**Supplementary material: online-only document**

1. Figure 1. Flow diagram outlining the identification and extraction of the TRD and MDD cohort (page 2)
2. Details of the services that TRD/MDD were referred to at the NHS Trust (page 3)
3. Table S1. Predictor variables for TRD status based on multistage analysis in binary logistic regression models (page 4)
4. Figure 2. The proportion of patient referrals across clinical services based on TRD status (page 5)
5. Table S2. Antidepressant prescriptions by number of treatment lines in the TRD cohort (page 6)
6. Table S3. Mental and physical health comorbid diagnoses by treatment resistance (page 7)
7. Semi-structured interview schedule for clinical staff (pages 8-9)
8. Semi-structured interview schedule for people with TRD (page 10)
9. Table S4. Summary of themes, subthemes and corresponding quotes from qualitative interviews with people with TRD and clinicians (pages 11-12)

**Stage 1:**

**Anonymised records** showing patients who are aged ≥18 years old and have a MDD diagnosis (F32-, F33, F34, F38).

**(n = 23, 357)**

**Stage 1:**

Available **EPMA data** for MDD cohort and identifying TRD status using Maudsley Prescribing guidelines

(n = 7, 396)

**Stage 2:**

Patients who hold a MDD diagnosis (just F32-F33) with no other excluding comorbidities

**(n = 5,136)**

**Stage 2:**

Excluded n = 2,260 who had either a current and/or historic comorbid diagnosis of: 1) bipolar disorder/mania; 2) psychosis-related; 3) dementia-related or 4) cognitive/neurological disorders. See exclusion criteria.

**MDD**

n = 2, 675

**TRD**

n = 2, 461

*Figure 1*. Flow diagram outlining the identification and extraction of the TRD and MDD cohort.

**Details of the services that people with TRD and MDD patients were referred to at the NHS Trust**

1. Community services (e.g. community mental health teams, liaison psychiatry)
2. Urgent care (e.g. crisis teams, accident and emergency)
3. Specialist services (e.g., psychology, other allied health professional contact and electroconvulsive therapy)
4. Inpatient services
5. Forensic services
6. Addictions services

| **Model** | **Predictor variables** |
| --- | --- |
| Model 1 | F32 (Major depressive disorder, single episode) vs F33 (Major depressive disorder, recurrent) at first recorded depression diagnosis, presence of psychotic symptoms linked to first recorded depression diagnosis, employment status (employed vs economically inactive) |
| Model 2 | Presence of mental health comorbidities: 1) Substance abuse disorders 2) Anxiety disorders and personality disorders |
| Model 3 | Recorded history of deliberate self-harm (no vs yes) |
| Model 4 | Presence of physical health comorbidities: 1) Cardiovascular diseases 2) Respiratory diseases 3) Gastrointestinal diseases |

**Table S1. Predictor variables for TRD status based on multistage analysis in binary logistic regression models**

Notes: All predictor variables were significantly different between groups (TRD vs MDD), see Tables 2 and 3.


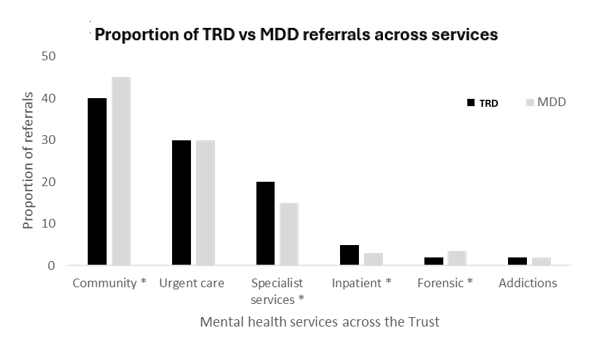


*Figure 2.* The proportion of patient referrals (N = 9,161) across clinical services based on TRD status. Main chi square test indicates a significant association in TRD vs MDD referrals across services (χ2(5)=92.68, *p*<0.001). * p<0.001, urgent care and addictions services p=0.840.

**Table S2. Antidepressant prescriptions by number of treatment lines in the TRD cohort**

| Number of treatment lines | N (%) |
| --- | --- |
| 2 | 946 (35.40) |
| 3 | 529 (19.78) |
| ≥4 | 986 (36.93) |

Notes: Percentages reflect the proportion of patients within the TRD cohort trialling 2, 3, or ≥4 lines of antidepressants during the current depressive episode. The total number of antidepressant prescriptions across all patients in the TRD cohort was 9,478.

|  | **2 lines of treatment** | **3 lines of treatment** | **≥ 4 lines of treatment** |  |
| --- | --- | --- | --- | --- |
|  | (N = 946) | (N = 529) | (N = 986) |  |
|  | N (%) | N (%) | N (%) | P |
| **Comorbid mental health diagnoses** |  |  |  |  |
| Substance Abuse Disorders – including abuse and dependence (yes; at least one of the below disorders)^a^ | 184 (19.45) | 120 (22.68) | 209 (21.20) | **<0.001** |
| Alcohol^b^ | 79 (8.35) | 52 (9.83) | 95 (9.64) | 0.992 |
| Drugs of abuse^c^ | 22 (2.33) | 18 (3.40) | 33 (3.35) | 0.045 |
| Sedatives or hypnotics^d^ | 2 (0.21) | 3 (0.57) | 5 (0.51) | 0.115 |
| Tobacco^e^ | 45 (4.76) | 55 (10.40) | 70 (13.74) | **<0.001** |
| Other/Multiple drugs^f^ | 13 (1.37) | 8 (1.51) | 13 (1.32) | 0.358 |
| Anxiety Disorders (yes; e.g. generalised anxiety, post-traumatic stress, social anxiety, panic disorder)^g^ | 224 (23.67) | 174 (32.90) | 361 (36.61) | **<0.001** |
| Eating Disorders (yes; e.g. anorexia, bulimia)^h^ | 11 (1.16) | 12 (2.27) | 16 (1.62) | 0.199 |
| Personality Disorders (yes; e.g. borderline personality disorder)^i^ | 100 (10.57) | 90 (17.01) | 217 (22.01) | **<0.001** |
| Intentional Self Harm^j^ | 16 (1.69) | 19 (3.59) | 31 (6.19) | **<0.001** |
| Psychotic illness^k^ | 107 (11.31) | 78 (14.74) | 185 (18.77) | **<0.001** |
| Developmental Disorders (yes; e.g. aspergers, autism)^l^ | 15 (1.59) | 12 (2.27) | 17 (1.72) | **<0.001** |
| **Comorbid physical health diagnoses** |  |  |  |  |
| Diabetes^m^ | 64 (6.77) | 49 (9.26) | 94 (9.53) | **<0.001** |
| Type 1 diabetes^n^ | 11 (1.16) | 13 (2.46) | 17 (1.72) | 0.070 |
| Type 2 diabetes^o^ | 48 (5.07) | 32 (6.05) | 65 (6.59) | **<0.001** |
| Unspecified diabetes^p^ | 5 (0.53) | 4 (0.76 | 12 (1.22) | 0.162 |
| Cardiovascular Diseases (yes; e.g. hypertension, ischemia, diseases of veins etc.)^q^ | 50 (5.29) | 36 (6.81) | 49 (4.97) | **<0.001** |
| Respiratory Diseases (yes; e.g. asthma, bronchitis, pneumonia etc.)^r^ | 35 (3.70) | 28 (5.29) | 30 (3.04) | **<0.001** |
| Gastrointestinal Diseases (yes; e.g. irritable bowel syndrome, ulcers, liver disease etc.)^s^ | 28 (2.96) | 36 (6.81) | 54 (5.48) | **<0.001** |

**Table S3. Mental and physical health comorbid diagnoses by treatment resistance**

Notes: ^a^Chi square = 42.11(1*),* p<0.001; ^b^Chi square = 0.10(1), p=0.992; ^c^Chi square = 8.04(1), p=0.045; ^d^Chi square = 5.93(1), p=0.115; ^e^Chi square = 89.56(1), p<0.001; ^f^Chi square = 3.23(1), p=0.358; ^g^Chi-square = 91.00(1), p<0.001; ^h^Chi square = 4.65(1), p=0.199; ^i^Chi square = 75.81(1), p<0.001; ^j^Chi square = 53.21(1), p<0.001; ^k^Chi square = 64.92(1*),* p<0.001; ^l^Chi-square = 22.90(1*),* p<0.001; ^m^Chi square = 30.83(1*),* p<0.001; ^n^: Chi square = 7.04(1*),* p=0.070; ^o^Chi square = 28.47(1*),* p<0.001; ^p^Chi square = 5.14(1*),* p=0.162; ^q^Chi square = 73.26(1*),* p<0.001; ^r^Chi square = 37.35(1*),* p<0.001; ^s^Chi square = 80.25(1*),* p<0.001

**Semi-structured interview schedule for the clinical staff**

1. What percentage of patients from your caseload would fit the description of treatment-resistant depression (TRD), defined as having had at least 2 trials of antidepressant therapy, at adequate dose and length?
   1. (prompts: How many patients do you see that have depression and out of those, how many would meet the criteria for TRD? What proportion of your whole caseload would classify as having TRD?)
   2. (prompts: Can you describe some of the difficulties you may have encountered when supporting people with TRD on your caseload e.g. difficulties within the Trust, difficulties with patients?)
2. How effective do you think the currently available treatments are for TRD in the Trust?
   1. (prompts: both biological and psychological. Can you give an example of a treatment that you offered that was successful for a patient and one that wasn’t so successful)
3. How do you think the treatment of TRD could be improved in the Trust
   1. (Prompts: Are there any treatments that you would like to see that aren’t currently offered within the Trust. Would complex mental health disorders such as TRD benefit from treatment that targets its comorbidities?)
   2. (prompts: Can you estimate the proportion of your TRD caseload that come to you with complex problems/comorbidities and to what extent is that a particular problem of treating TRD?)
4. What would help facilitate your clinical decision making and management of TRD patients?
   1. (prompts: What added support would be useful in the management of TRD patients e.g. input from specific clinicians, more evidence-based treatments being offered within the Trust, better awareness of TRD across the Trust in both patients and clinicians?)
5. Are there specific treatments (biological and/or psychological) that you think would benefit TRD patients on your caseload?

**Semi-structured interview schedule for people with TRD**

1. Can you talk me through your experience of having depression?
   1. (prompts: when did you first think you might be depressed – (note ascertain how long they have struggled with depression) other mental health disorders they may have experienced alongside their depression)
2. Can you talk me through your experiences of seeking help for your depression?
   1. (prompts: did you contact any clinicians, if so, who/how many? What treatment options were you offered at this point (i.e. offered but not necessarily tried) and how long were you waiting before you were offered your first treatment.
3. What treatments have you had so far to help treat your depression?
   1. (prompts: What biological treatments have you tried (e.g., medication); What psychological treatments have you tried; Have you tried any alternative therapies?)
4. What difficulties or barriers have you experienced with these treatments?
   1. (potential follow-up question: in your own words, can you describe what it feels like to have tried these treatments and for them to not helped you?)
5. How has your TRD and treatment experiences affected other areas of your life?
   1. (prompts: such as your relationships, your ability to work; financial wellbeing?)
6. What are the important factors that you would want to see as part of a new care pathway for TRD?
   1. (prompts: specific treatments you may have heard about? timely assessment? Specific clinician’s input?)
7. Would you be interested in taking part in any new TRD related research studies in the Trust? (please explain your answer)

**Table S4. Summary of themes, subthemes and corresponding quotes from qualitative interviews with people with TRD and clinicians**

| Theme | Sub-themes |  | Participant | Mixed-methods inferences |
| --- | --- | --- | --- | --- |
| TRD criteria | Lack of awareness around TRD as an indicator of severity and treatment resistance  TRD diagnostic criteria |  | I have seen patients who have been on like every single antidepressant under the sun. And when you go back and look at the records … they might have had some sort of response, but then it wasn't titrated up. (C2) | The discrepancy in awareness and understanding of TRD adds to the challenges associated with undertreatment of this condition. |
| Experiences | Experiences of people with TRD  Experiences of clinicians |  | It's a difficult one when you try treatment, and it doesn't work. It made me feel worse about myself then actually better, they kept upping the dose I was just getting to the point where I just don't feel any better. (P4) | Patients expressed frustration with their inability to “fight-off” their depression, inadequate treatment responses, limited treatment options, and the profound impact on their lives, affecting work, relationships, and independence (e.g., being able to drive). |
| Current treatment pathway | Biological treatments  Psychological treatments |  | I did like one session of CBT and in that session, they basically were like yeah CBT is not gonna cut it for you … so I believe I self-referred for that (psychological therapy) but that was quite a while ago, its hard to remember but I went to that kind of therapy for a couple years. It helped somewhat but I don’t think it really approached the kind of core of the issue as it were. (P2) | Both patients and clinicians advocated for more personalised approaches rather than taking a ‘one size fits all’ approach, stressing the need to address functional aspects through services such as occupational therapy (OT) and criticised treatment prioritisation based on risk levels, neglecting those without immediate risks. Moreover, there was consensus on the need for better access to psychological treatments, despite extended wait times for assessments and treatments. |
| Barriers to treatment | Institutional barriers/organisational barriers  TRD-related illness barriers |  | Psychiatric services are really under the threshold for people with really enduring, almost dangerous mental health conditions, you know psychosis bipolar type disorders I’ve worn myself out because I find if I don’t go to work-I don’t even get out of bed … I probably won’t shower and won’t get out of bed or answer the door. (P1) | Patients highlighted the lack of accessible information regarding TRD within the Trust, emphasising the need for resources such as leaflets explaining and outlining the condition and its treatment options. Long waiting periods, especially for psychology services, were identified as significant barriers, often resulting in hospital admissions due to lack of timely care. |
| Facilitators to treatment | Positive experiences  Engaging in TRD research |  | Because I don’t want anybody to have to suffer in the same way that I have and if it’s any improvement-I would offer anything really-to make it better. (P3) | Patients eagerly sought information on TRD research within the Trust, feeling reassured by efforts of clinicians and researchers to advance the treatment and management of TRD. |
| Future treatment pathway recommendations | Patient recommendations  Clinician recommendations |  | A strategy, I mean whether that just be something as simple as a flow chart once it's diagnosed, this is the available support to go here, here, or here… to have some visible support mechanism in terms of where to steer for treatment, who to contact would be the helpful thing. (C6) | To enhance the care and support for TRD patients, both patients and clinicians recommended a multifaceted approach to the management and treatment of the condition. |
